# Sweet Corrosion Inhibition by CO_2_ Capture

**DOI:** 10.3390/molecules27165209

**Published:** 2022-08-16

**Authors:** Jesus Porcayo-Calderon, Jorge Canto, L. M. Martinez-de-la-Escalera, Adrian Neri

**Affiliations:** 1 Department of Chemical Engineering and Metallurgy, University of Sonora, Hermosillo 83000, Mexico; 2 Corrosion y Proteccion (CyP), Buffon 46, Mexico City 11590, Mexico

**Keywords:** corrosion, CO_2_ capture, lanthanum chloride, lanthanum carbonate, inhibitor

## Abstract

The most practical and economical way to combat the problems derived from CO_2_ corrosion (sweet corrosion) is the use of corrosion inhibitors of organic origin. Its main protection mechanism is based on its ability to adsorb on the metal surface, forming a barrier between the metal surface and the aggressive medium. However, despite its excellent performance, its inhibition efficiency can be compromised with the increase in temperature as well as the shear stresses. In this study, the use of an inorganic inhibitor is proposed that has not been considered as an inhibitor of sweet corrosion. The reported studies are based on using LaCl_3_ as a corrosion inhibitor. Its behavior was evaluated on 1018 carbon steel using electrochemical measurements, such as potentiodynamic polarization curves, open-circuit potential measurements, linear polarization resistance measurements, and electrochemical impedance. The results showed an inhibition efficiency of the sweet corrosion process greater than 95%, and that the inhibition mechanism was different from the classic corrosion process in CO_2_-free electrolytes. In this case, it was observed that the inhibitory capacity of the La^3+^ cations is based on a CO_2_-capture process and the precipitation of a barrier layer of lanthanum carbonate (La_2_(CO_3_)_3_).

## 1. Introduction

CO_2_ corrosion is one of the main causes of the degradation of metallic materials in the oil industry and is the cause of significant environmental damage, as well as economic damage due to the unavailability of equipment. This degradation process is a surface process that encouraged an in-depth study of the mechanisms of metallic dissolution [1,2,3] as well as how to mitigate it.

The degradation of materials by dissolved CO_2_, also known as sweet corrosion, is a process that causes damage to the internal surface of the pipelines used in the transportation of hydrocarbons. This type of damage is also known as internal corrosion. The pipelines used to transport hydrocarbons are made of carbon steel, where its main element (Fe) has a standard potential that places it as a highly active element in acid conditions. Although carbon steel can protect itself due to the precipitation on its surface of a layer of iron carbonate (FeCO_3_), the conditions for this to occur are environments of high temperature, alkaline pH, and high concentration of Fe^2+^ cations [3,4,5], in addition to the high availability of dissolved CO_2_. However, the high concentration of Fe^2+^ cations implies an advanced corrosion process so that self-sealing can occur.

Due to the impossibility of carrying out a continuous inspection to determine the internal surface state, one of the main actions used to inhibit the metal dissolution process has been the injection of corrosion inhibitors into the corrosive fluid. These compounds can form protective layers on the metal surface, either by absorption or precipitation. Most corrosion inhibitors are organic compounds, and in their structure, they contain nitrogen (N), sulfur (S), and oxygen (O) atoms, unsaturated functional groups, as well as aromatic rings that favor their adsorption on the metal surface, due to a lone pair of electrons [6,7,8,9,10,11]. Corrosion inhibitors can reduce metal dissolution by at least 90% when they are added to the electrolyte in doses as low as ppm. However, it has been reported [12,13,14] that its inhibition efficiency decreases with increasing temperature.

In recent decades, there has been great interest in using rare earth elements to synthesize compounds with corrosion inhibitory capacity in aggressive environments [15,16,17,18,19,20,21,22,23,24,25]. Its excellent corrosion inhibiting ability has been attributed to the high reactivity of rare earth. During the electrochemical corrosion process, the cathodic reaction activates the O_2_ reduction reaction, causing an increase in pH at the cathodic sites [22]. These conditions favor the precipitation of rare earth ions on the metal surface, forming a protective film that decreases the cathodic reaction rate and, therefore, the corrosion current density [16,18,19,22,26,27].

Based on the above, the objective of this research work is to study the effect of an inorganic compound (LaCl_3_) on the corrosion behavior of carbon steel in a solution of NaCl (3.5% by weight) saturated with CO_2_ at 60 °C. LaCl_3_ can be considered a safe compound since its toxicity is similar to that of NaCl [16,27,28], and to date, it has not been considered a corrosion inhibitor in environments with dissolved CO_2_. The test temperature was chosen to reference the experimental conditions under which the performance of corrosion inhibitors with potential use in pipelines used to transport hydrocarbons is evaluated. The average temperature of the transported fluid is 50–60 °C [7,8,9,10]. The inhibitory capacity of LaCl_3_ was determined through electrochemical studies, such as polarization curves, measurements of open circuit potential, polarization resistance, and electrochemical impedance, as well as complementary analyses using scanning electron microscopy techniques (SEM-EDS) and X-ray diffraction.

## 2. Results and Discussion

### 2.1. Potentiodynamic Polarization Curves

**Figure 1:**
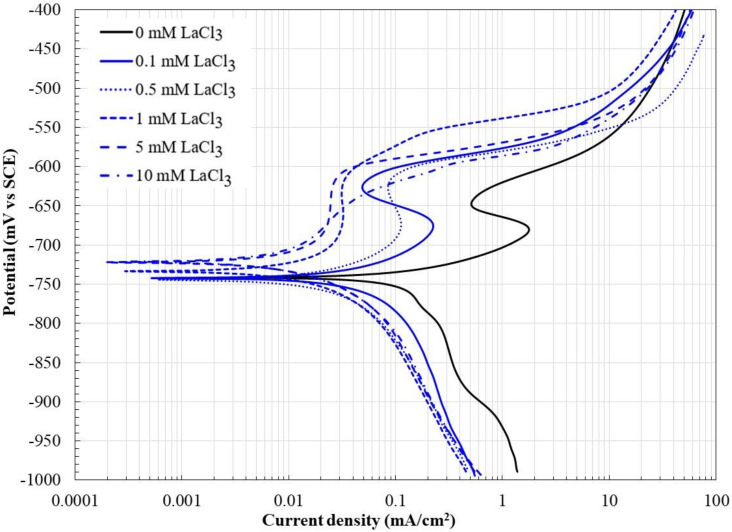
Polarization curves for 1018 carbon steel in CO_2_-saturated brine at different concentrations of lanthanum chloride after 50 h of immersion.

Figure 1 shows the potentiodynamic polarization curves of carbon steel 1018 after 50 h of immersion in a 3.5% NaCl solution saturated with CO_2_ at 60 °C with and without the addition of LaCl_3_ as a corrosion inhibitor.

In the absence of a corrosion inhibitor, the oxidation reaction of Fe determines the behavior of the anodic branch. The observed anodic behavior corresponds to that suggested by increasing the polarization of the anodic branch [3,29]—that is, initially an active dissolution behavior followed by a transition behavior, a pre-passive behavior, and finally a passive state. The extension of these zones depends on the electrolyte composition where the Fe is immersed.

The addition of the inhibitor caused the displacement of the polarization curves towards lower corrosion densities and slightly nobler potentials. A decrease in the cathodic current density and the development of a pseudo-passive zone in the anodic branch are noticeable. These changes suggest that the presence of La^3+^ ions caused a decrease in both the anodic and cathodic reaction rates, possibly due to the development of a La-based protective film on the metal surface. In the scientific literature, there is little information on the use of t rare earth or their compounds as corrosion inhibitors in electrolytes with dissolved CO_2_. The use of organic Pr compounds as corrosion inhibitors has been reported [30], and an increase in corrosion potential and an effect on both the anodic and cathodic branches have also been indicated. The observed behavior corresponds to a mixed-type inhibition; similar behavior has been reported with the addition of compounds based on rare earth in environments rich in chlorides [16,17,18,23,24].

Table 1 shows the values of the electrochemical parameters obtained from the Tafel zones of the potentiodynamic polarization curves (Figure 1).

In the absence of an inhibitor, it was observed that the anodic slope is of the same order as those reported for metal dissolution processes exclusively [4], and its value is in good agreement with that obtained by the expression (46 mV_exp_ vs. 44 mV_calc_) [3]:

(1) $$b_{a}=\frac{2.303RT}{\left(1+\beta \right)F},$$

where *R* is the gas constant, *T* is the temperature (K), *β* is the symmetry coefficient (*β* = 0.5), and *F* is the Faraday constant. This suggests that even after 50 h of immersion in the electrolyte, the carbon steel was not able to develop a protective layer on its surface. The decrease in current density observed in the anodic branch between −685 and −650 mV may correspond to the precipitation of an amorphous colloid (FeCO_3_/Fe(HCO_3_)_2_) due to the supersaturation of Fe^2+^ ions, as has been suggested in other studies [4].

It is observed that at inhibitor concentrations less than 1.0 mM, the corrosion potential remains practically constant, and at concentrations greater than 0.5 mM, the corrosion potential shifts slightly towards the noble direction. Similarly, in the presence of the inhibitor, the slope of the anodic branch increases, which indicates a decrease in the rate of metal dissolution. On the contrary, in general, the slope of the cathodic branch decreased, possibly due to a decrease in the cathodic sites that caused a decrease in the rate of the cathodic reaction. It has been suggested that when the Tafel slope changes and does not follow a trend according to the concentration of the added inhibitor, it simply acts as a barrier reducing the reaction area [10]. The Icorr decreased, up to an order of magnitude, with increasing inhibitor concentration up to 1.0 mM, and at higher concentrations, it tends to increase slightly. The increase in Icorr values at concentrations greater than 1.0 mM may be due to the increase in Cl^−^ ion concentration due to the addition of the inhibitor (LaCl_3_) [18,23,24]. The few reported studies indicate that the decrease in the corrosion rate due to the addition of compounds based on rare earth is greater in environments saturated by CO_2_ than in aerated environments, possibly due to the formation of different protective films [30].

### 2.2. Open Circuit Potential Measurements

**Figure 2:**
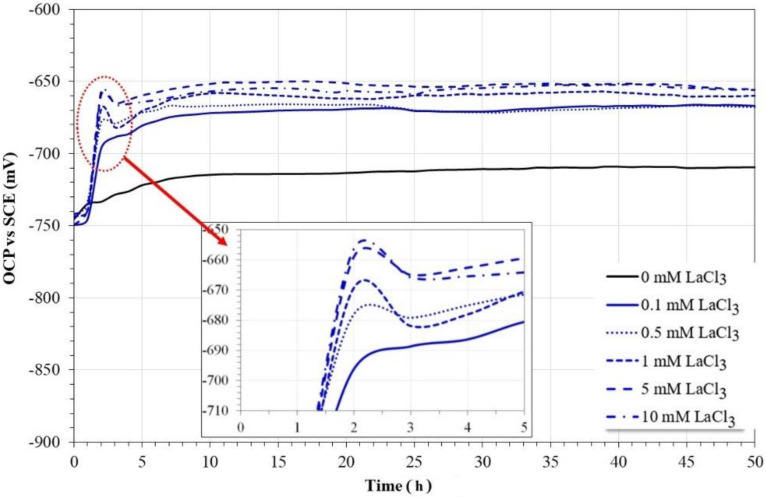
Evolution of OCP values for 1018 carbon steel in CO_2_-saturated brine at different concentrations of lanthanum chloride.

Figure 2 shows the variation in OCP values for 1018 carbon steel during 50 h of immersion in a 3.5% NaCl solution saturated with CO_2_ at 60 °C with and without the addition of LaCl_3_ as a corrosion inhibitor.

In the absence of an inhibitor, carbon steel showed an increase in its OCP values up to the first 10 h of immersion, and subsequently, its values tended to increase slowly until reaching a steady state at the end of the test. In this case, the observed trend may correspond to the formation and accumulation of corrosion products on steel surfaces. On the other hand, in the presence of the inhibitor, in all cases after its addition, a sudden increase in OCP values was observed, followed immediately by a slight decrease. Subsequently, a slow increase in OCP values was observed until reaching the steady state after 10 h of immersion.

In general, abrupt changes in the OCP values have been associated with a reduction or increase in the rate of the anodic and/or cathodic processes [16,17]. In particular, the shifts to more active potentials, in addition to indicating an increase in the metal dissolution rate, also suggest a process of inhibition of the cathodic reaction [16], such as that observed with the use of compounds based on rare earth as corrosion inhibitors in solutions rich in chlorides [16,23,24,31]. This suggests that in solutions rich in chlorides saturated with CO_2_, the mechanism of inhibition of rare earth is different from that observed in the absence of CO_2_.

The sudden increase in OCP values may be associated with a change in solution chemistry and/or the formation and partial dissolution of a protective film on the steel surface. Notwithstanding the evident shift in OCP values, the magnitude of the shift is less than 85 mV, and this suggests that lanthanum chloride acts as a mixed inhibitor [32,33].

### 2.3. Linear Polarization Resistance Measurements

**Figure 3:**
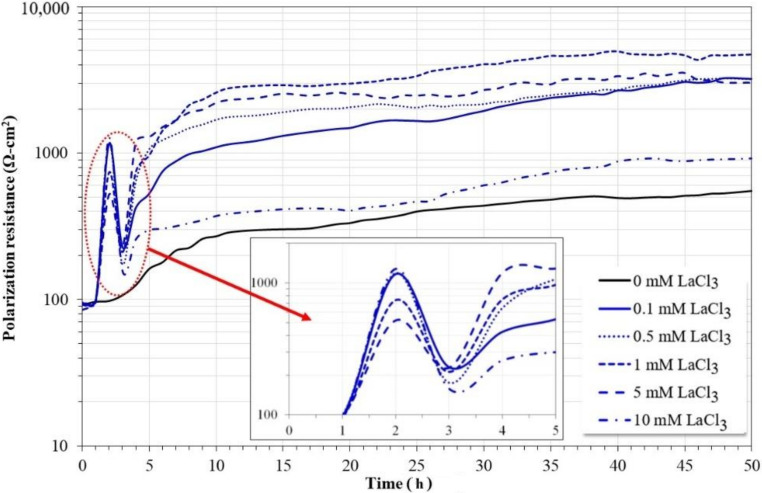
Evolution of polarization resistance values for 1018 carbon steel in CO_2_-saturated brine at different concentrations of lanthanum chloride.

Figure 3 shows the variation in polarization resistance values for carbon steel 1018 during 50 h of immersion in a 3.5% NaCl solution saturated with CO_2_ at 60 °C with and without the addition of LaCl_3_ as a corrosion inhibitor.

In the absence of an inhibitor, carbon steel showed a rapid increase in its Rp values in the first 10 h of immersion, and subsequently, the trend is maintained but at a slower rate until the end of the test. The observed trend may correspond to a rapid corrosion process and accumulation of corrosion products on the steel surface, which limited the available reaction area. However, in the presence of the inhibitor, a similar behavior was observed regardless of its concentration. In all cases, immediately after the addition of the inhibitor, an abrupt increase in Rp values was observed, followed by an abrupt decrease. After this behavior, the Rp values tended to increase rapidly until 10 h of immersion, and then a constant increase was observed until the end of the test. The initial behavior is consistent with that observed with the OCP values (Figure 2).

This new evidence suggests that the addition of the inhibitor may have caused an instantaneous disturbance in the chemical composition of the solution, thereby causing a temporary decrease in its corrosivity. The magnitude of the Rp values is consistent with the Icorr values observed with the polarization curves (Figure 1); that is, the Rp values increase with the concentration of the inhibitor up to 1.0 mM, and at higher concentrations, they tend to decrease, possibly due to the increase in the concentration of Cl^−^ ions [18,23,24].

**Figure 4:**
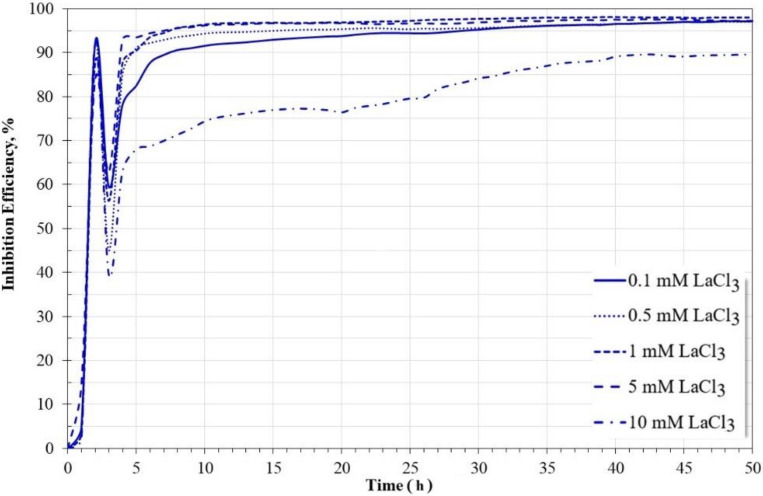
Effect of LaCl_3_ concentration on inhibition efficiency.

Figure 4 shows the variation in the inhibition efficiency of LaCl_3_ as a function of its concentration for carbon steel immersed in a 3.5% NaCl solution saturated with CO_2_ for 50 h. The calculations show that the inhibition efficiencies are greater than 95% for concentrations up to 5 mM. At higher concentrations, it tends to decrease, possibly due to the increase in the concentration of Cl^−^ ions. The maximum inhibition efficiency was 98% for a concentration of 1 mM of LaCl_3_. The results show that LaCl_3_ is a more efficient corrosion inhibitor in environments rich in chlorides saturated by CO_2_ than in the absence of CO_2_ [23,24], possibly because its protective action is different.

### 2.4. Electrochemical Impedance Spectroscopy Measurements

**Figure 5:**
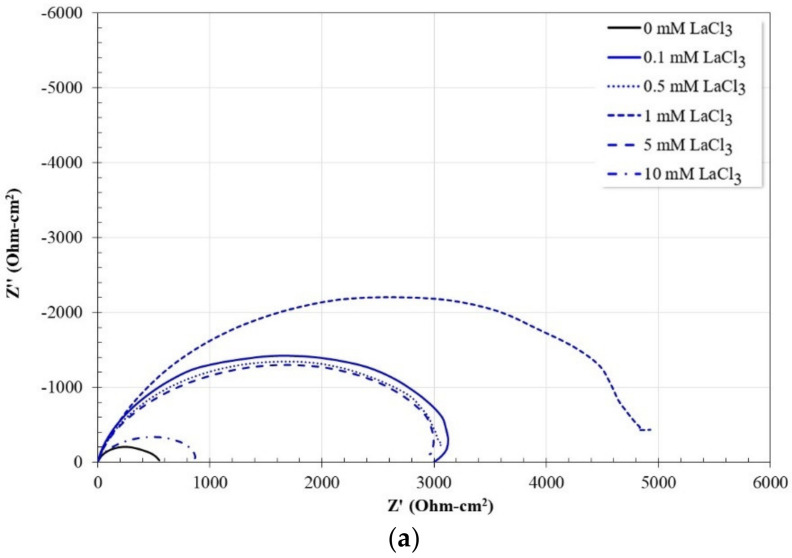

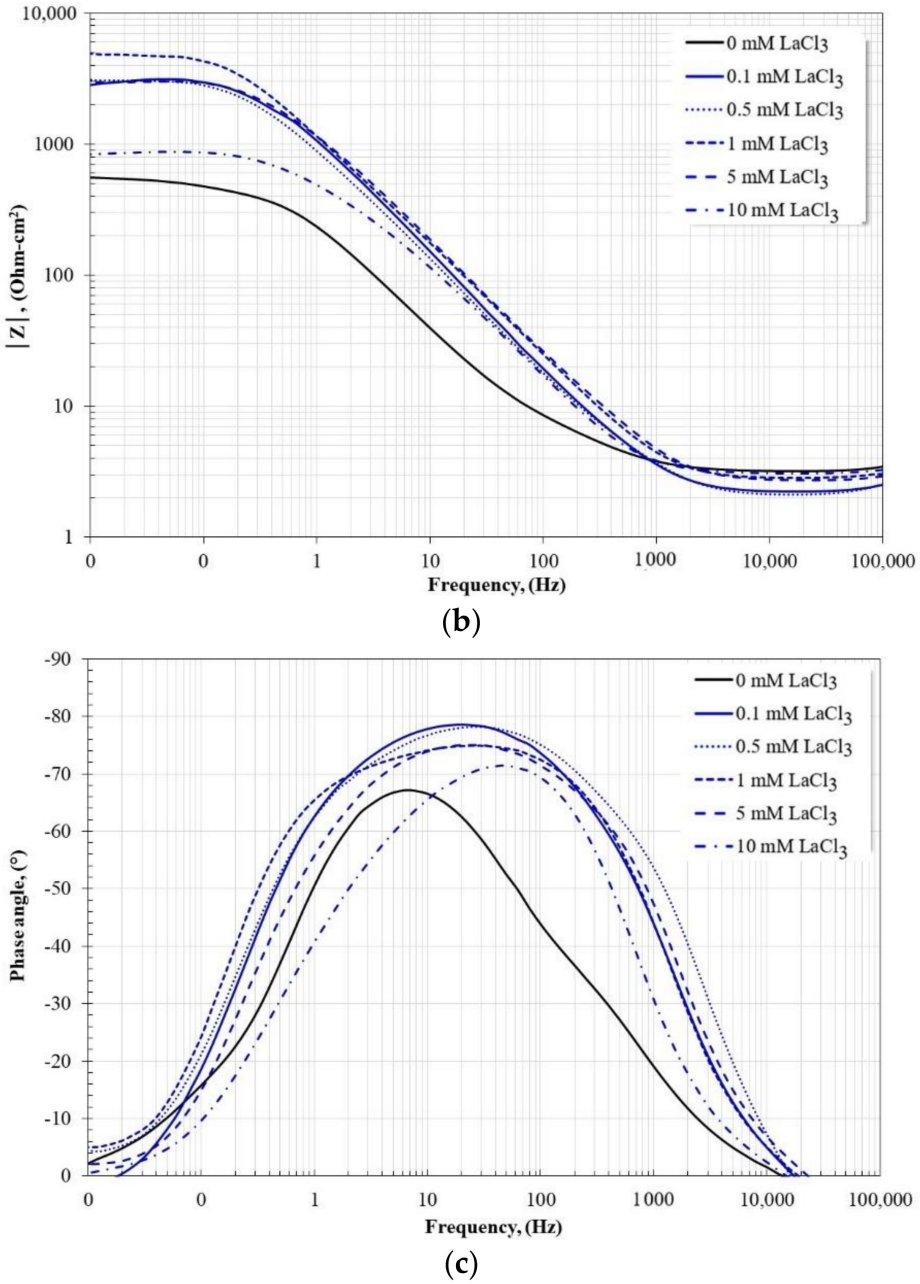
Nyquist and Bode diagrams for 1018 carbon steel in CO_2_-saturated brine at different concentrations of lanthanum chloride after 50 h of immersion. (**a**) Nyquist diagram; (**b**) Bode diagram in its impedance modulus format; (**c**) Bode diagram in its phase angle format.

Figure 5 shows the Nyquist and Bode diagrams for carbon steel after 50 h of immersion in a 3.5% NaCl solution saturated with CO_2_ at 60 °C with and without the addition of LaCl_3_ as a corrosion inhibitor.

From the impedance spectra, it is observed that all the spectra show similar behavior. The Nyquist diagram shows the apparent presence of a single capacitive semicircle whose maximum diameter is obtained for an inhibitor concentration of 1 mM. From the Bode plot in its impedance modulus format, the presence of the high and low-frequency plateau is observed, as well as a linear log f-log |Z| relationship in the intermediate frequency region. The evolution of the low-frequency plateau is consistent with the magnitude of the diameter of the capacitive semicircles. From the Bode plot in its phase angle format, the presence of only one time constant is confirmed in all cases. However, in the presence of an inhibitor, the magnitude of the phase angle is bigger, and its location is at higher frequencies than that observed in the absence of an inhibitor.

**Figure 6:**
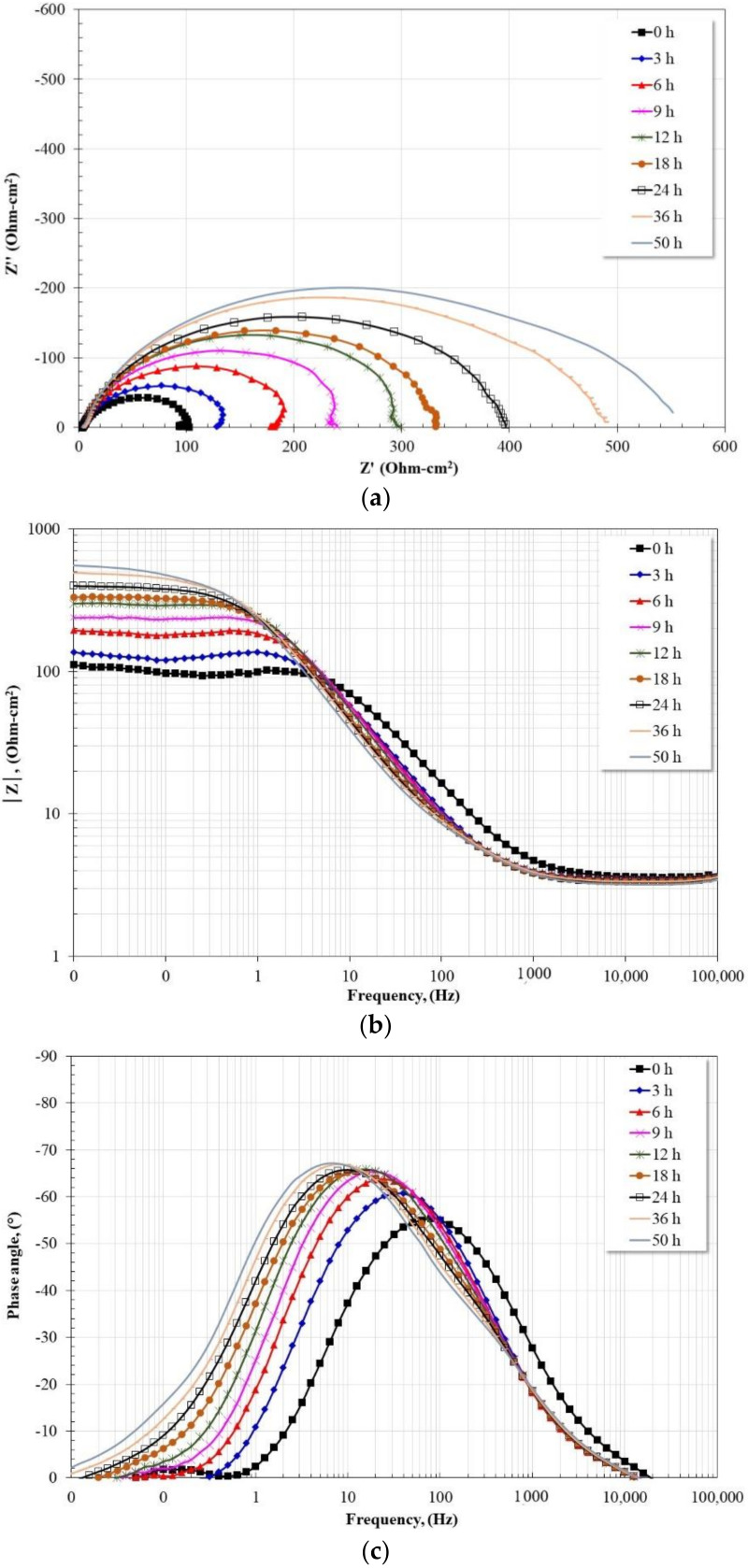
Evolution of Nyquist and Bode diagrams for 1018 steel in CO_2_-saturated brine at 60 °C. (**a**) Nyquist diagram; (**b**) Bode diagram in its impedance modulus format; (**c**) Bode diagram in its phase angle format.

**Figure 7:**
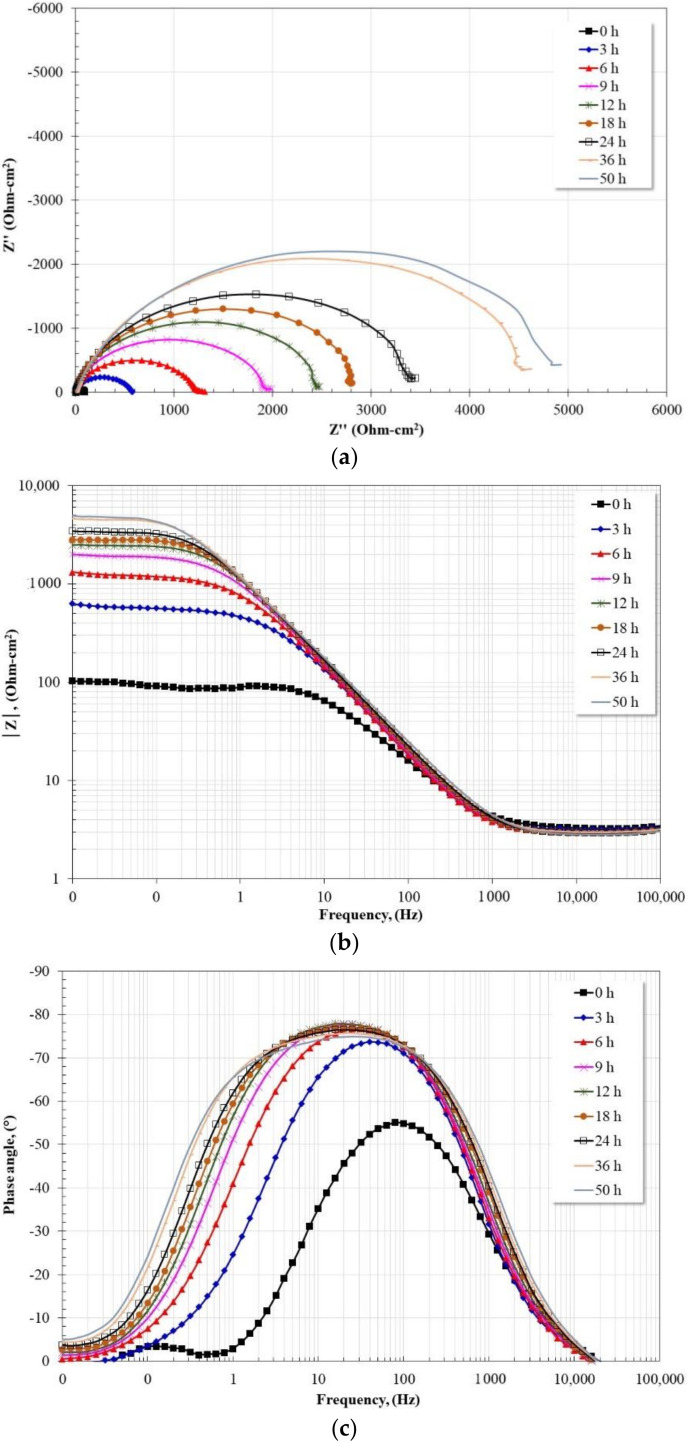
Evolution of Nyquist and Bode diagrams for 1018 carbon steel in CO_2_-saturated brine at 60 °C and 1 mM of LaCl_3_. (**a**) Nyquist diagram; (**b**) Bode diagram in its impedance modulus format; (**c**) Bode diagram in its phase angle format.

Since in the presence of the inhibitor, the impedance spectra showed a similar evolution, in subsequent analyses, only the evolution of the spectra in the absence (Figure 6) and presence of the inhibitor at a single concentration, 1 mM LaCl_3_ (Figure 7) is presented.

The evolution of the impedance spectra for the case of carbon steel in the absence of an inhibitor based on the Nyquist diagram shows the presence of a depressed capacitive semicircle, whose diameter increases as a function of immersion time. Data scatter in the low-frequency region has been associated with inhomogeneity and microroughness of the working electrode surface [11,34,35]. From the Bode plot in its impedance modulus format, |Z|, in the high-frequency region, the presence of the high-frequency plateau from 1000 Hz is observed, as well as the development of a linear relationship, log |Z|-log f, which extends to the low-frequency region as a function of time. In addition, in the low-frequency region, the presence of the low-frequency plateau is observed, the length of which decreases and moves to higher impedance modulus values with immersion time. From the Bode plot in its phase angle format, the presence of a time constant is visible, which shifts towards lower frequencies (80 Hz 🡪 5 Hz) as a function of the immersion time, in addition, its maximum phase angle increases in the same way (55° 🡪 67°). The characteristics observed are congruent with a material whose surface forms a layer of corrosion products with protective characteristics.

**Figure 8:**
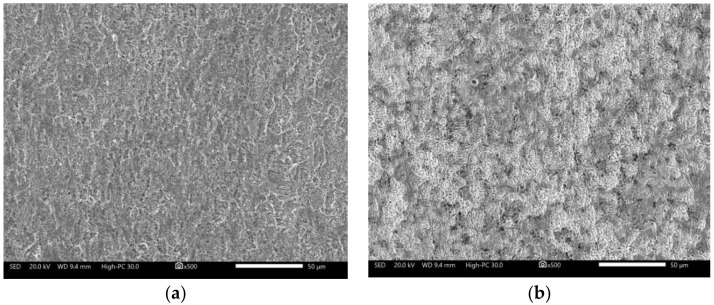
The morphological aspect of 1018 carbon steel after corrosion test in CO_2_-saturated brine solution after 50 h: (**a**) corrosion products surface; (**b**) clean surface.

However, after the corrosion test, the surface characteristics of the carbon steel (Figure 8) showed the accumulation of a thin layer of corrosion products. Under it, a heterogeneous attack with the presence of pitting was observed. Then, the increase in the diameter of the capacitive semicircle, impedance module and phase angle may be associated with the accumulation of a porous layer of corrosion products that decreased the reaction area but not the corrosion process [19].

**Figure 9:**
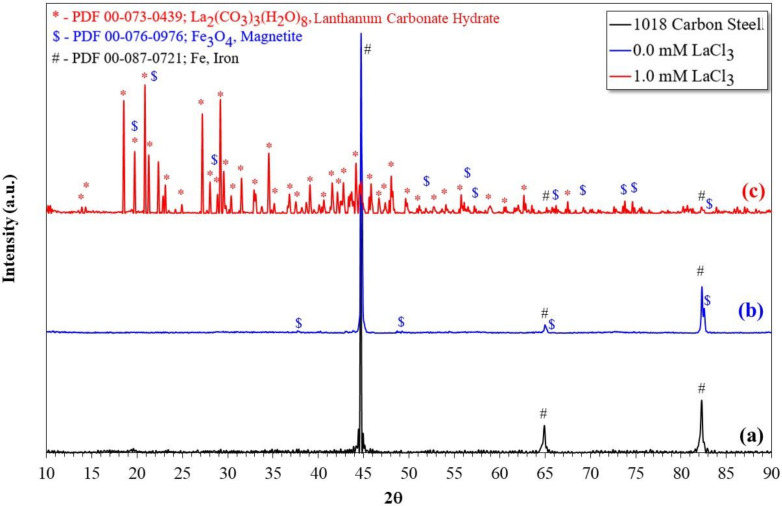
X-ray diffraction pattern of the surface of 1018 carbon steel; (**a**) not corroded, (**b**) corroded, (**c**) corroded with the addition of LaCl_3_.

In this type of environment, the main corrosion product with protected characteristics that develop on the surface of carbon steel is iron carbonate, FeCO_3_. However, the XRD analysis performed on the surface of the samples (Figure 9) did not detect their presence. This is because its formation is favored at higher temperatures and longer immersion times than those evaluated here [36]. In this case, the main corrosion product detected was Fe_3_O_4_.

In general, the corrosion process of carbon steel in deaerated conditions is due to the following anodic and cathodic reactions:

(2) $$\mathrm{Fe}↔{\mathrm{Fe}}^{2+}+2e^{-},$$

(3) $$2H^{+}+2e^{-}↔H_{2},$$

During the active dissolution process, the Fe^2+^ cations can participate in secondary reactions that favor the formation of corrosion products, which can act as a barrier to the free access of the electrolyte and, with it, a reduction in the rate of metal dissolution.

(4) $${\mathrm{Fe}}^{2+}+2{\mathrm{OH}}^{-}↔\mathrm{Fe}{\left(\mathrm{OH}\right)}_{2}+H_{2}O,$$

(5) $$4\mathrm{Fe}{\left(\mathrm{OH}\right)}_{2}+O_{2}+2H_{2}O↔4\mathrm{Fe}{\left(\mathrm{OH}\right)}_{3},$$

(6) $$4\mathrm{Fe}{\left(\mathrm{OH}\right)}_{2}+O_{2}↔2{\mathrm{Fe}}_{2}O_{3}+4H_{2}O,$$

(7) $$\mathrm{Fe}{\left(\mathrm{OH}\right)}_{3}+O_{2}↔\mathrm{FeOOH}+H_{2}O,$$

(8) $$4\mathrm{Fe}{\left(\mathrm{OH}\right)}_{2}+O_{2}↔4\mathrm{FeOOH}+2H_{2}O,$$

(9) $$2\mathrm{FeOOH}+H_{2}O↔{\mathrm{Fe}}_{2}O_{3}+H_{2}O,$$

On the other hand, the evolution of the impedance spectra in the presence of the inhibitor (in the entire range of concentrations) presented a behavior like that shown in Figure 7. From the Nyquist diagram, the presence of a capacitive semicircle whose diameter increases with immersion time is shown. The magnitude of the diameter increase is greater than that observed in the absence of the inhibitor. From the Bode plot in its impedance modulus format, |Z|, the formation of the high-frequency plateau was observed from 1000 Hz, and in the intermediate frequency region, a linear relationship, log |Z|-log f, with only one slope was observed. In the low-frequency region, the presence of the low-frequency plateau was observed, which moves to higher impedance modulus values as a function of time. The magnitude of its displacement is greater than that observed in the absence of the inhibitor. From the Bode plot in its phase angle format, the presence of a single time constant is visible, which shifts towards lower frequencies (80 🡪 20 Hz) and increases its maximum phase angle (55° 🡪 78°) as a function of time. The magnitude of this shift was less than in the absence of an inhibitor, and the increase in phase angle maximum was greater. These characteristics suggest the formation of a thicker protective layer with higher capacitive properties than that formed in the absence of the inhibitor.

**Figure 10:**
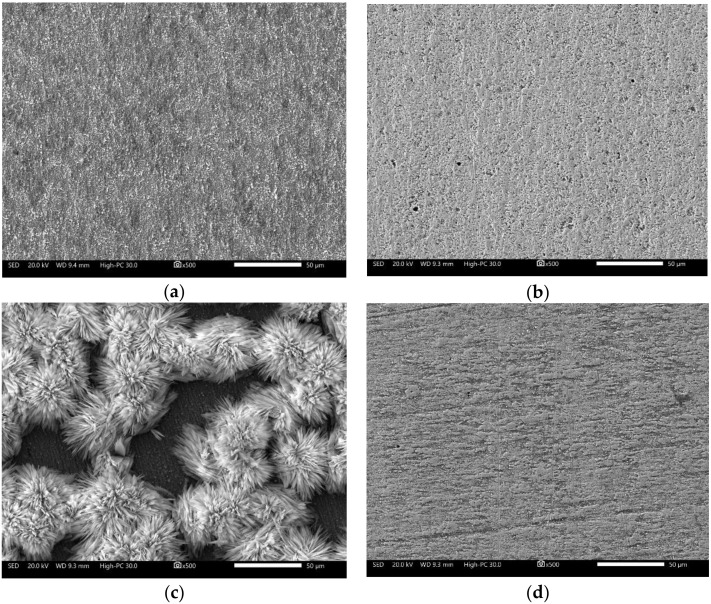

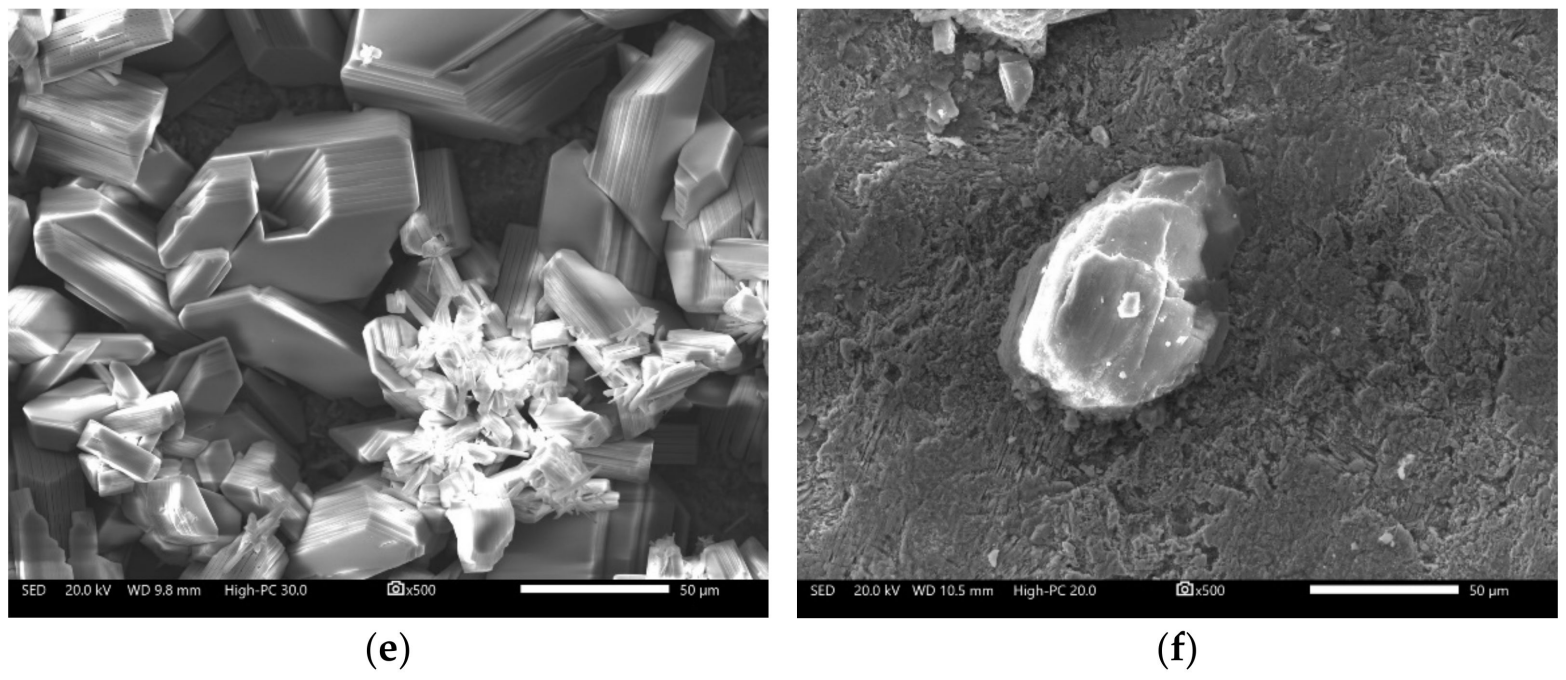
The morphological aspect of 1018 carbon steel after corrosion test in CO_2_-saturated brine solution at different concentrations of the added inhibitor. 0.1 mM; (**a**) corrosion products surface; (**b**) clean surface. 1.0 mM; (**c**) corrosion products surface; (**d**) clean surface. 10.0 mM; (**e**) corrosion products surface; (**f**) clean surface.

The surface characteristics of the carbon steel (Figure 10) after the corrosion test showed the presence of precipitates whose appearance changes depending on the concentration of the added inhibitor. At low concentrations (0.1 mM), a surface with a layer of reaction products of dense appearance was observed; when increasing the concentration (1.0 mM), the formation of structures that resemble the chrysanthemum flower was observed, and at higher concentrations (10.0 mM), the precipitates were different in shape and of higher density. To observe the appearance of the surface, the precipitates formed were removed by immersion in a HCl solution (10% *v*/*v*) for 5–10 s. The removal of the superficial layers showed that at low concentrations of the inhibitor, a more homogeneous surface was obtained than that observed in the absence of the inhibitor, and when increasing its concentration, scratch marks of the surface preparation were still observed. However, in the evaluation of the highest concentration of the inhibitor, an irregular surface with the presence of firmly adhered remnant deposits was observed, possibly due to the increase in the concentration of Cl^−^ ions due to the addition of the inhibitor.

**Figure 11:**
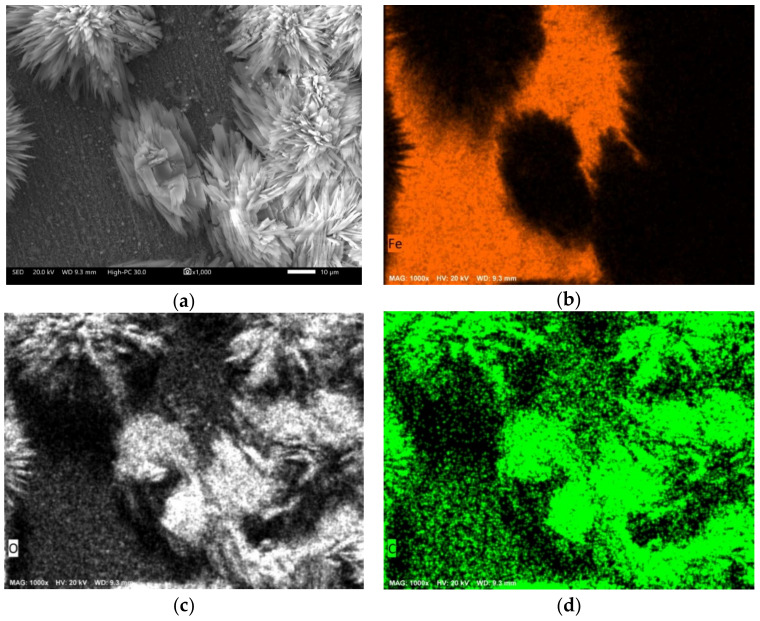

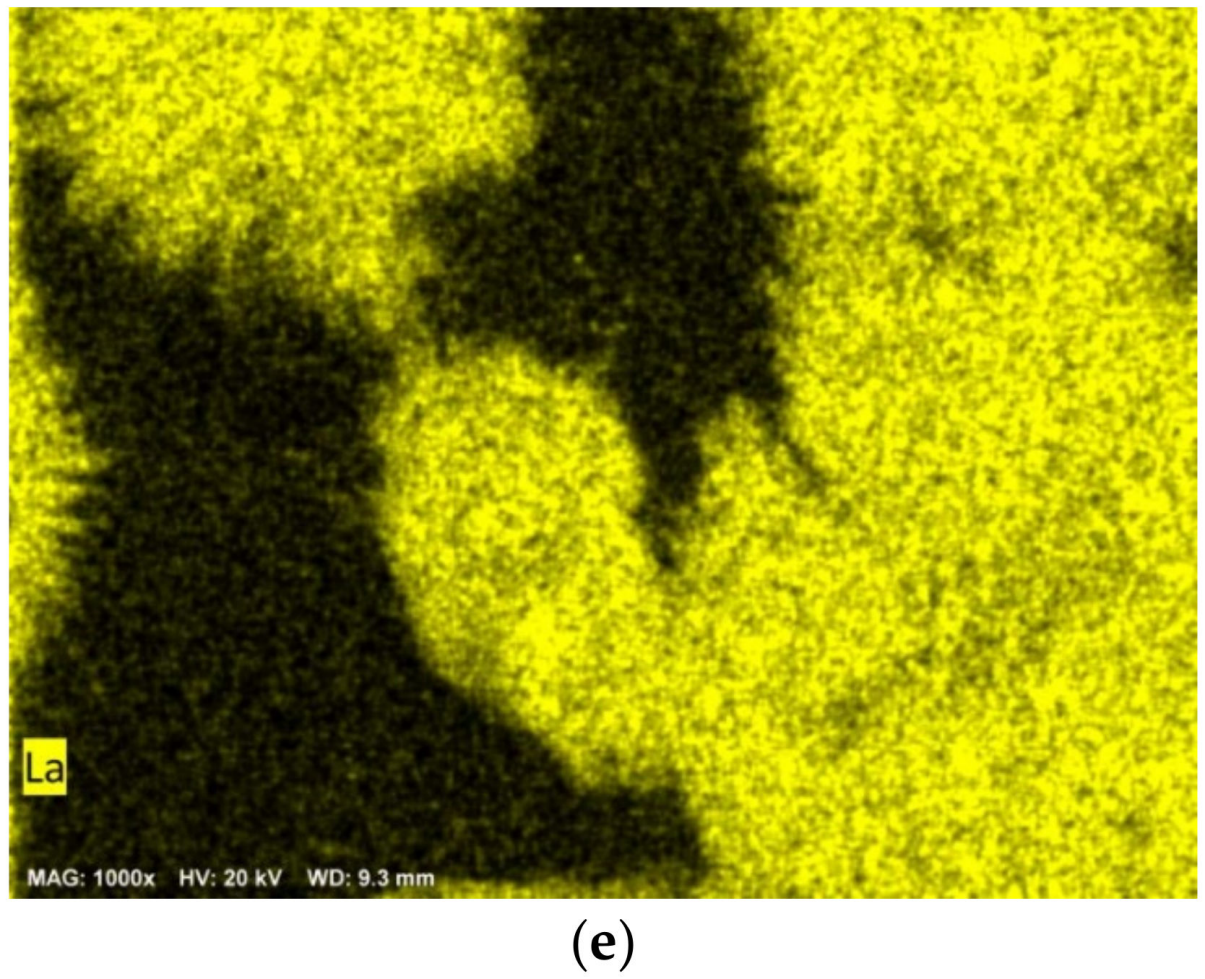
Approach to the surface of 1018 carbon steel evaluated with the addition of 1.0 mM of LaCl_3_. (**a**) secondary electron image; (**b**) Fe mapping; (**c**) O mapping; (**d**) C mapping; (**e**) La mapping.

An EDS analysis and element mapping (Figure 11) revealed that the formed deposits are associated with La-O-C and that over the areas where the bulky deposits did not form, there was also this type of compound in the form of thin films.

XRD analysis of the carbon steel surface (Figure 9) showed that the precipitated La-O-C compound corresponds to lanthanum carbonate (La_2_(CO_3_)_3_). Regardless of the concentration of the inhibitor, lanthanum carbonate was the main compound detected in all cases. Its presence is associated with dissolved CO_2_ and La^3+^ cations, the product of inhibitor dissociation. Then, the increase in the diameter of the capacitive semicircle, impedance module and phase angle corresponds to the precipitation of this compound, which acted as a barrier layer between the metallic surface and the electrolyte.

### 2.5. CO_2_-Capture and Protection Mechanism

Unlike the multiple corrosion studies in CO_2_-free electrolytes where it has been reported that the protection mechanism of rare earth is due to their precipitation (as oxides and hydroxides) by hydrolysis reactions of their metallic cations with the OH^−^ ions produced in the cathodic sites [19,23,24,25,37,38,39,40], a different mechanism was observed in this study, namely, CO_2_ capture and precipitation of lanthanum carbonate (La_2_(CO_3_)_3_) as a protective barrier.

Recent studies [41] indicate that in environments with CO_2_, the formation of rare earth carbonates, Ln_2_(CO_3_)_3_, is thermodynamically more favorable than species such as Ln(OH)_3_, Ln_2_O_3_, Ln(OH)^−4,^ and even Ln^3+^. This is because its free energy of formation (ΔG°_f_) is considerably more favorable. This indicates that lanthanum carbonates are more stable species, and therefore their protective capacity is greater. In addition, it is reported that the formation of rare earth carbonates is more favorable in aqueous systems with available carbonate ions (CO_2_^−3^) and that this mechanism corresponds to a CO_2_ capture process, where the CO_2_ capture efficiency is more than 95% [42].

Based on the above, the behavior observed in the OCP and RPL measurements can be justified, that is, the abrupt increase in both the open circuit potential and the polarization resistance when LaCl_3_ was added. A rapid capture process of the dissolved CO_2_ present in the vicinity of the metal surface occurred, thereby reducing the corrosivity of the electrolyte. The subsequent increase in these parameters (OCP, LPR) was a consequence of the constant bubbling of CO_2_, which restored its concentration. Because the CO_2_ hydration process is a slow step [3,5], the restoration of OCP and RPL values did not occur instantaneously. These sudden changes show the high reactivity of the La^3+^ cations, which offers a significant advantage over organic inhibitors. Under real conditions, it has been observed that the maximum effectiveness of organic inhibitors is detected up to two months after continuous injection. [43]

The capture process of CO_2_ and its precipitation as lanthanum carbonate can be explained according to the following reactions [41]:

(10) $${\mathrm{LaCl}}_{3_{\left(s\right)}}↔{\mathrm{La}}_{\left(\mathrm{aq}\right)}^{3+}+3{\mathrm{Cl}}_{\left(\mathrm{aq}\right)}^{-},$$

(11) $${\mathrm{CO}}_{2_{\left(g\right)}}+{\;H}_{2}O_{\left(l\right)}↔H_{2}{\mathrm{CO}}_{3_{\left(\mathrm{aq}\right)}},$$

(12) $$H_{2}{\mathrm{CO}}_{3_{\left(\mathrm{aq}\right)}}↔{\mathrm{HCO}}_{3_{\left(\mathrm{aq}\right)}}^{-}+H_{\left(\mathrm{aq}\right)}^{+},$$

(13) $${\mathrm{HCO}}_{3_{\left(\mathrm{aq}\right)}}^{-}↔{\mathrm{CO}}_{3_{\left(\mathrm{aq}\right)}}^{2-}+H_{\left(\mathrm{aq}\right)}^{+},$$

(14) $$2{\mathrm{La}}_{\left(\mathrm{aq}\right)}^{3+}+3{\mathrm{CO}}_{3_{\left(\mathrm{aq}\right)}}^{2-}↔{\mathrm{La}}_{2}{\left({\mathrm{CO}}_{3}\right)}_{3_{\left(s\right)}},$$

During the metal dissolution process, the dissociation reaction of carbonic acid (H_2_CO_3_), responsible for the formation of the electrochemically active species (H^+^) that buffers the concentration of hydrogen ions on the metal surface [1,2], favors the formation of carbonate ions and with it the precipitation reaction of lanthanum carbonate. However, because carbonic acid is a weak diprotic acid, it has been suggested that the first dissociation step is a fast process, and the second is a slow process favored around neutral pH. Therefore, it is possible that the precipitation of lanthanum carbonate can also occur in a similar way to the precipitation reaction of iron carbonate in acid environments [5]:

(15) $$2{\mathrm{La}}_{\left(\mathrm{aq}\right)}^{3+}+3{\mathrm{HCO}}_{3_{\left(\mathrm{aq}\right)}}^{-}↔{\mathrm{La}}_{2}{\left({\mathrm{HCO}}_{3}\right)}_{3_{\left(s\right)}},$$

(16) $${\mathrm{La}}_{2}{\left({\mathrm{HCO}}_{3}\right)}_{3_{\left(s\right)}}↔{\mathrm{La}}_{2}{\left({\mathrm{CO}}_{3}\right)}_{3_{\left(s\right)}}+3H^{+},$$

The hydration and dissociation reactions of the dissolved CO_2_ are reactions inherent in the classic mechanism of the sweet corrosion process [1,2,3], and in this case, the presence of the La^3+^ cations interrupt this route by capturing the dissolved CO_2_, thereby reducing the corrosivity of the electrolyte, and increases the corrosion resistance of the steel by forming a protective layer of La_2_(CO_3_)_3_ on its surface.

The source of cations for the formation of these carbonates does not depend on the Fe^2+^ cations generated during the corrosion process, so their formation is favored at lower temperatures. Although temperature influences inhibition efficiency [12,13,14,15], in this case, a decrease in solution temperature will increase the amount of dissolved CO_2_ [44] available to react with La^3+^ cations; therefore, its inhibition efficiency will not be affected in a significant way. In addition, for each La_2_(CO_3_)_3_ molecule formed, three moles of C are captured, unlike the formation of FeCO_3_, whose ratio is 1:1. The formation and precipitation of lanthanum carbonate increases both the adsorption and hydration of CO_2_, thus favoring a CO_2_-capture process and transforming it into a more stable product with low solubility [45]. This reduces the corrosiveness of the electrolyte, favoring the formation of a barrier and thereby reducing the corrosion rate of carbon steel. When CO_2_ capture occurs by its dissolution in water, it is also called a CO_2_ mineralization process [41,46].

Furthermore, lanthanum carbonate has a higher free energy of formation and greater E–pH stability range than iron carbonate [47]. The formation of iron carbonate is favored at temperatures and times greater than those evaluated here [4]. In this way, the formation and precipitation of a barrier layer of lanthanum carbonate guarantees a greater protective capacity.

## 3. Materials and Methods

As a study material, 1018 carbon steel specimens with dimensions 10 × 10 × 5 mm were used (1 cm^2^ reaction area). Using the spot-welding technique, a copper conductor wire was welded to one of the faces of the specimens, and in this condition, they were encapsulated in epoxy resin. The encapsulated samples were roughened with abrasive paper from grade 120 to grade 600. Subsequently, they were washed with distilled water and ethanol, dried, and used immediately in the corrosion tests.

As corrosive medium, a solution of NaCl (3.5% by weight) saturated with CO_2_ at a temperature of 60 °C was used. The solution was saturated with CO_2_ for at least one hour before starting the tests. To maintain saturation, CO_2_ bubbling was maintained for the duration of the tests performed. Lanthanum chloride (LaCl_3_) was used as a corrosion inhibitor at concentrations of 0.0001 M, 0.0005 M, 0.001 M, 0.005 M, and 0.01 M. In all cases, the inhibitor was added to the corrosive medium one hour after the immersion of the working electrode. The inhibitor was added in its pure state, and its homogenization in the electrolyte was carried out with stirring for 5–10 s.

The electrochemical tests were carried out in a three-electrode cell where the encapsulated specimens were the working electrodes, the reference electrode was a saturated calomel electrode, and a graphite bar was used as a counter electrode. The electrochemical tests carried out were potentiodynamic polarization curves, open circuit potential (OCP) measurements, linear polarization resistance (RPL), and electrochemical impedance spectroscopy (EIS). Before each test, the working electrode was immersed in the electrolyte for 15 min to achieve a steady state. Electrochemical measurements were performed for 50 h with a GAMRY potentiostat/galvanostat (model 1000).

The potentiodynamic polarization curves were obtained by polarizing the working electrode ±300 mV with respect to the corrosion potential at a scanning speed of 1 mV/s. OCP measurements were made at hourly intervals. RPL measurements were made by polarizing the working electrode ±10 mV with respect to its open-circuit potential at hourly intervals. EIS measurements were obtained by applying to the working electrode an amplitude perturbation of 10 mV (AC) with respect to the open-circuit potential over a frequency range of 100 kHz to 0.01 Hz.

The inhibition efficiency was calculated according to the following expression (Rp_i_ = resistance to polarization in the presence of an inhibitor, Rp_b_ = resistance to polarization in the absence of an inhibitor):

(17) $$E\left(\%\right)=\left(\frac{Rp_{i}-Rp_{b}}{Rp_{i}}\right)\times 100,$$

The surfaces of the samples were analyzed by SEM-EDS with an acceleration voltage of 20 kV, and by X-ray diffraction in the range of 10° ≤ 2θ ≤ 90° with a step of 0.003° and a time of 320 s per step.

## 4. Conclusions

Electrochemical studies carried out show that lanthanum chloride shows a high sweet corrosion inhibition capacity at concentrations as low as 1 mM. Its inhibition efficiency is greater than 95% and is greater than that observed in CO_2_-free electrolytes. Its addition to the electrolyte caused a rapid increase in the OCP and RPL values due to a decrease in the electrolyte corrosivity due to the capture of dissolved CO_2_. At higher concentrations, its inhibition efficiency tends to decrease due to the increase in the concentration of Cl^−^ ions. Electrochemical impedance studies showed that the precipitation and deposition of lanthanum carbonate causes an increase in the capacitive response of the carbon steel surface and therefore, an increase in the charge transfer resistance.

Unlike the classical inhibition mechanisms, where the inhibitor molecules are adsorbed on the metal surface or precipitate on it by reacting with the OH^−^ ions generated at the cathodic sites, in this study, it was found that the La^3+^ cations react with CO_2_ dissolved forming a barrier layer on the metal surface. La^3+^ cations act by a CO_2_ capture process that interrupts the classic route of the sweet corrosion mechanism, unlike organic inhibitors, whose inhibition mechanism is based on their adsorption capacity on the metal surface.

## Figures and Tables

**Table 1:** Electrochemical parameters of the polarization curves.

LaCl_3_	Ecorr	ba	bc	Icorr
(mM)	(mV)	(mv/Dec)	(mV/Dec)	(mA/cm_2_)
0	−740	46	379	0.158
0.1	−742	112	363	0.099
0.5	−744	174	204	0.052
1.0	−733	176	228	0.013
5.0	−722	131	244	0.031

## Data Availability

Not applicable.
